# Supplementary material for: Association between oral microbiome and seven types of cancers in East Asian population: a two-sample Mendelian randomization analysis
Source: Front Mol Biosci. 2023 Nov 21;10:1327893. doi: 10.3389/fmolb.2023.1327893 (PMC10702768; doi:10.3389/fmolb.2023.1327893)
Supplement: Supplementary file 1 [file DataSheet1.ZIP › Supplementary Materials/MR plots of saliva/saliva═╝/Breast cancer/pheno.484_to_breast cancer_forest.pdf]

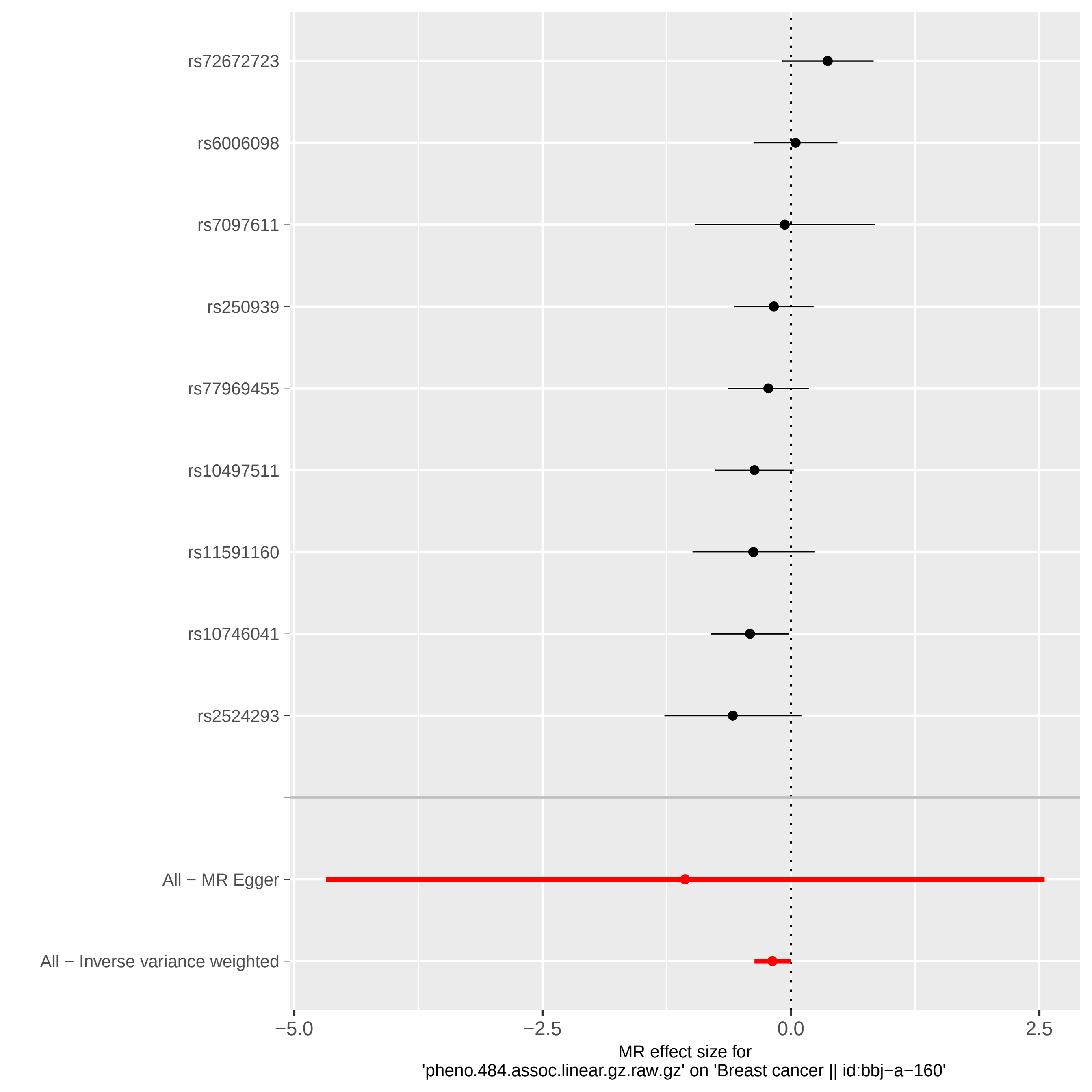
rs72672723
rs6006098
rs7097611
rs250939
rs77969455
rs10497511
rs11591160
rs10746041
rs2524293
All − MR Egger
All − Inverse variance weighted
−5.0
−2.5
0.0
2.5
MR effect size for
'pheno.484.assoc.linear.gz.raw.gz' on 'Breast cancer || id:bbj−a−160'
